# Editorial: Lactic acid bacteria and their bioactive compounds: key regulators of gut microbiota and immune function

**DOI:** 10.3389/fmicb.2025.1720803

**Published:** 2025-10-31

**Authors:** Sabina Fijan, Nathalie Connil, Bojana Bogovič Matijašić, Marikunte Yanjarappa Sreenivasa, Marimuthu Anandharaj

**Affiliations:** ^1^Faculty of Health Sciences, University of Maribor, Maribor, Slovenia; ^2^Université de Rouen Normandie, Université de Caen Normandie, Normandie Université, Research Unit Bacterial Communication and Anti-Infectious Strategies (CBSA, UR4312), Rouen, France; ^3^Biotechnical Faculty, Department of Animal Science, Institute of Dairy Science, University of Ljubljana, Domžale, Slovenia; ^4^Department of Studies in Microbiology, University of Mysore, Mysuru, Karnataka, India; ^5^Lawrence Berkeley National Laboratory, U.S. Department of Energy Joint Genome Institute, Berkeley, CA, United States

**Keywords:** lactic acid bacteria, probiotics, gut microbiota, health, bacteria, fungi

## 1 Introduction

The gut microbiota is the complex and dynamic community of bacteria, fungi, parasites, and viruses that enter a symbiotic relationship with the cells of the host. They protect the host against pathogens, shape and strengthen the intestinal epithelium and influence the immune system (Calo-Mata et al., 2016; Cheng et al., 2019; Hou et al., 2022). The bacterial metabolites can enter the host bloodstream and influence other parts of the body through various axes in humans and animals, such as gut-microbiota-brain, gut microbiota-skin, gut-vagina, gut-liver, gut-bones etc (Chakrabarti et al., 2022; Gasaly et al., 2021; Kim et al., 2024). However, many aspects of modern lifestyle can cause alterations in the gut microbiota and lead to numerous diseases including inflammatory bowel disease, diabetes mellitus, obesity, and metabolic syndrome (Agus et al., 2021; Khan et al., 2019). Probiotics and other beneficial microbes can exert many beneficial effects on their hosts by modulating of the gut microbiota. While the term probiotic is reserved only for well-characterized strains with clinically proven health benefits, other beneficial microbes include microbes responsible for the fermentation of foods, such as yogurt, kefir, kombucha, kimchi, and many others that have been shown to have various health benefits (Hill et al., 2014; Marco et al., 2017, 2021). Many of these beneficial microbes belong to lactic acid bacteria.

**Figure 1:**
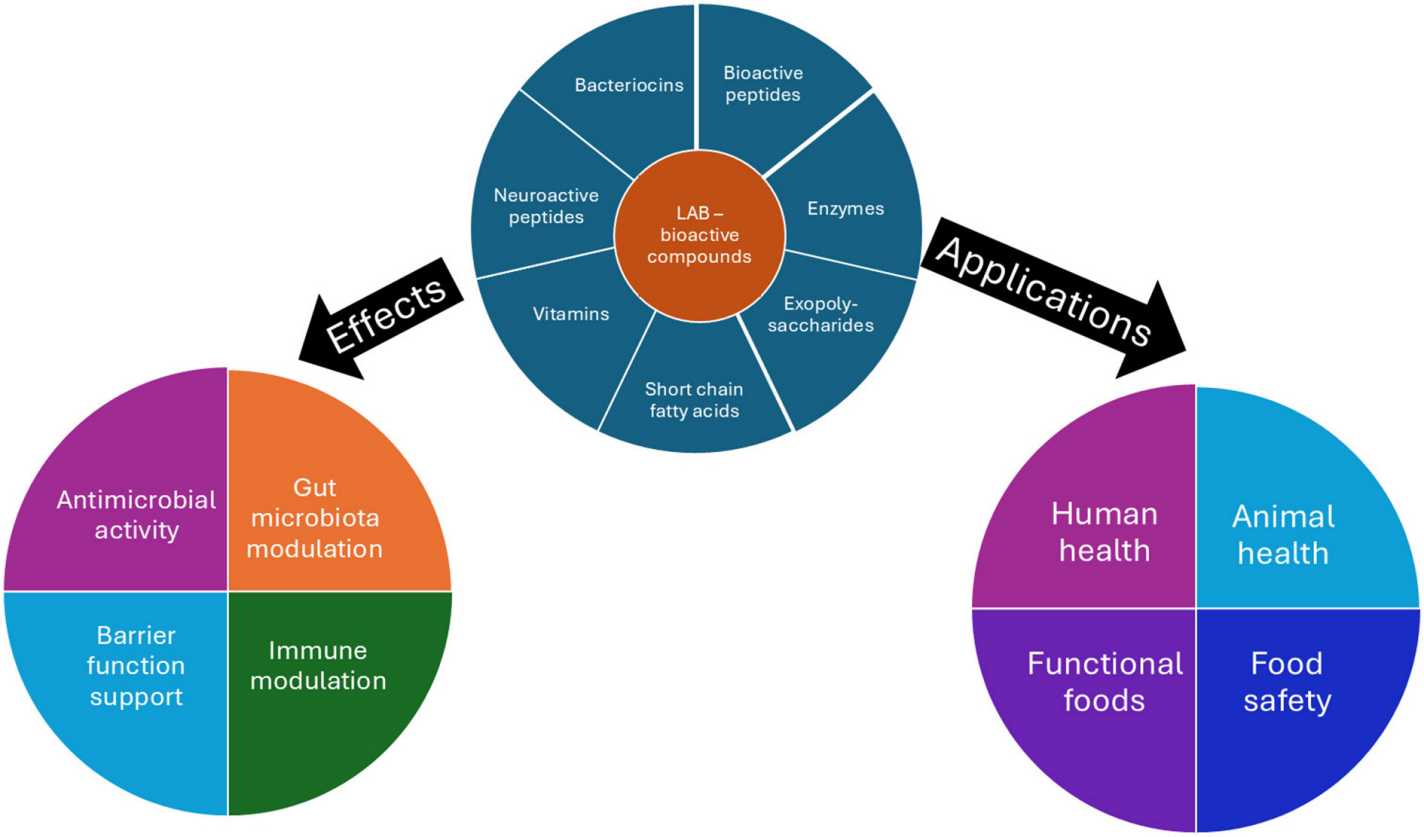
Microbial bioactives: mechanisms and applications in human, animal, and food health.

Lactic acid bacteria (LAB) are Gram-positive, usually non-motile, catalase-negative, aerotolerant rods or cocci with high tolerance to low pH that produce lactic acid by fermentation of carbohydrates. Lactic acid bacteria have many therapeutic and functional properties that are beneficial to human health. Homofermentative LAB mainly produce lactic acid from hexose sugars. Facultative heterofermentative also ferment glucose to produce lactic acid, but can also ferment pentose sugars to acetic acid, ethanol, and formic acid. Heterofermentative LAB produce lactic acid and ethanol or acetic acid and carbon dioxide (Bintsis, 2018; Sharma et al., 2020). According to the new taxonomic note some of the main genera include *Lactobacillus, Limosilactobacillus, Lacticaseibacillus, Lactiplantibacillus, Levilactobacillus, Furfurilactobacillus, Acetilactobacillus, Lactococcus, Pediococcus, Leuconostoc, Streptococcus, Enterococcus, Oenococcus, Aerococcus, Carnobacterium, Vagococcus, Weissella* (Zheng et al., 2020). The mechanisms and applications in human, animal, and food health of the microbial bioactives are noted in Figure 1.

This Research Topic aimed to compile articles focusing on new insights into the role of lactic acid bacteria in the regulation of the gut microbiota. The main objectives included were understanding how LAB influence gut microbiota composition, identifying bioactive compounds produced by LAB and their effects, elucidating the molecular mechanisms underlying LAB's impact on immunity, pathogen exclusion, and intestinal barrier function, and exploring potential therapeutic interventions involving LAB. By addressing these questions, the research was aimed to fill existing gaps in our knowledge and provide a comprehensive understanding of LAB's multifaceted roles in gut health and beyond.

## 2 Overview of contributions

Several articles highlighted the capacity of LAB to influence lipid metabolism. Liang et al. isolated *Lactiplantibacillus plantarum* L-27-2, *Pediococcus lactis* L-14-1, and *Enterococcus faecium* from cats, reporting cholesterol-lowering and anti-inflammatory effects in mice. Yao et al. demonstrated that *Lacticaseibacillus paragasseri* HM018 derived from breast milk improved lipid and bile acid metabolism in hypercholesterolaemic rats, while Kumari et al. described the hypoglycaemic and hypolipidaemic effects of *Levilactobacillus brevis* RAMULAB54 from fermented sugarcane juice through activation of PPAR-γ. Together with Wang et al., who showed improved lipid metabolism in broilers following *Lactococcus lactis* subsp. *lactis* G423 supplementation, these studies reinforce the idea that LAB play an active role in host metabolic regulation. These findings confirm earlier reports that SCFAs and bile salt hydrolase activity of LAB are crucial in lipid homeostasis (Gil-Rodríguez and Beresford, 2021; Li et al., 2023).

Immune regulation was another major theme. Tang et al. demonstrated that *Weissella confusa* Wc1982 alleviated colitis in mice by suppressing pro-inflammatory cytokines while enriching *Akkermansia muciniphila*. Chen et al. dissected molecular pathways, showing that *Lacticaseibacillus reuteri* SBC5-3 suppressed NF-κB and MAPK signaling in porcine epithelial cells. Li et al. explored the synergistic role of berberine, which increased the number of *Lactobacillus Verrucomicrobia, Bacteroides*, and *Akkermansia* in a murine colitis model. These observations support a broader consensus that probiotics modulate both innate and adaptive immunity, reducing inflammation and enhancing barrier integrity (Mazziotta et al., 2023; Mercado-Monroy et al., 2025).

Several studies examined LAB in livestock and poultry, underscoring their role as antibiotic alternatives. Hou et al. showed that *Lactiplantibacillus plantarum* supplementation improved growth performance in chicks under reduced protein diets, while Zhao et al. found that *Ligilactobacillus salivarius* S10 improved growth and intestinal health in pigeons. Murakami et al. demonstrated reduced *Campylobacter jejuni* colonization in poultry following *Limosilactobacillus ingluviei* C37 supplementation, highlighting food safety implications. Yang, Shang et al. added evidence that *Ligilactobacillus salivarius* CGMCC17718 enhanced antioxidative capacity in heat-stressed mice. Together, these studies point toward LAB as tools for sustainable farming, with benefits extending to animal welfare, productivity, and reduced pathogen transmission (Raman et al., 2022).

Beyond the strains themselves, metabolites were key players. Yang, Liu et al. investigated reuterin, converted by *Limosilactobacillus reuteri* ATCC55730 showing efficient antimicrobial activity against *Escherichia coli* and *Salmonella* Typhimurium, while Koc et al. illustrated how sourdough fermentation with LAB enriched SCFA profiles and beneficial taxa during colonic fermentation. Such findings confirm that microbial metabolites mediate host signaling, influencing pathways ranging from gut motility to systemic inflammation (Hosseinkhani et al., 2021; Margolis et al., 2021).

The translational potential of LAB and their compounds is vast. As Zhang et al. discussed, modulation of gut microbiota, could influence leukemia development, suggesting that probiotics especially lactobacilli strains, may complement oncology research. More broadly, this aligns with the One Health concept, where LAB contribute not only to human health but also to animal health, sustainable agriculture, and food innovation.

However, challenges remain. Strain-specificity is critical—not all LAB conferred the same benefits, and clinical validation is needed. Also, personalized responses to probiotics emphasize the complexity of host–microbe interactions and suggest that future research should focus on precision nutrition and microbiome-informed interventions (Gibbons et al., 2022; Zmora et al., 2018).

## 3 Conclusion

The contributions in this Research Topic collectively confirm LAB as central regulators of gut microbiota and immune function. Through their bioactive compounds, LAB show lipid-lowering, immunomodulatory, and antimicrobial effects, with implications for human and animal health. Future studies need to connect molecular understanding, clinical testing, and practical application of LAB.
